# Advancement of Compositional and Microstructural Design of Intermetallic γ-TiAl Based Alloys Determined by Atom Probe Tomography

**DOI:** 10.3390/ma9090755

**Published:** 2016-09-06

**Authors:** Thomas Klein, Helmut Clemens, Svea Mayer

**Affiliations:** Department of Physical Metallurgy and Materials Testing, Montanuniversität Leoben, Roseggerstr. 12, 8700 Leoben, Austria; helmut.clemens@unileoben.ac.at (H.C.); svea.mayer@unileoben.ac.at (S.M.)

**Keywords:** titanium aluminides based on γ-TiAl, atom probe tomography, alloy design, microstructure formation, interfacial segregation, phase stability, hardening effects, site-specific specimen preparation

## Abstract

Advanced intermetallic alloys based on the γ-TiAl phase have become widely regarded as most promising candidates to replace heavier Ni-base superalloys as materials for high-temperature structural components, due to their facilitating properties of high creep and oxidation resistance in combination with a low density. Particularly, recently developed alloying concepts based on a β-solidification pathway, such as the so-called TNM alloy, which are already incorporated in aircraft engines, have emerged offering the advantage of being processible using near-conventional methods and the option to attain balanced mechanical properties via subsequent heat-treatment. Development trends for the improvement of alloying concepts, especially dealing with issues regarding alloying element distribution, nano-scale phase characterization, phase stability, and phase formation mechanisms demand the utilization of high-resolution techniques, mainly due to the multi-phase nature of advanced TiAl alloys. Atom probe tomography (APT) offers unique possibilities of characterizing chemical compositions with a high spatial resolution and has, therefore, been widely used in recent years with the aim of understanding the materials constitution and appearing basic phenomena on the atomic scale and applying these findings to alloy development. This review, thus, aims at summarizing scientific works regarding the application of atom probe tomography towards the understanding and further development of intermetallic TiAl alloys.

## 1. Introduction

Intermetallic titanium aluminides belong to the most promising materials to meet today’s most prevalent demands for structural high-temperature materials of combining high strength with low density (next to other important characteristics like high creep and oxidation resistance as well as high modulus and strength retention at elevated temperatures) [1,2,3]. Due to these properties, TiAl alloys are particularly suitable for rotating components of modern propulsion systems, such as turbine blades of aero engines and turbocharger wheels of automotive engines. Especially, tight emission reduction targets, triggered by an increasing global ecological awareness [4], as directed by national and international initiatives and legislation [5,6], have become a major driving force in the development of this alloy class. Incorporation of innovative lightweight components into latest generation propulsion systems results in substantial reductions of greenhouse gases, CO_2_ and NO_x_, as well as fuel consumption. Consequently, extensive fundamental research projects in close collaboration with industrial partners have been launched to create the basis for knowledge-based compositional and microstructural design and concomitant feasible manufacturing routes. Today, titanium aluminides have become an established material class and sophisticated processing routes are available reflected by an increasing market penetration of TiAl components in both aerospace and automotive industry. Application examples are cast turbine blades of the low-pressure turbine introduced in the GEnx™ by General Electrics [7] and forged turbine blades used in the low-pressure turbine of the recently inaugurated Geared Turbofan™ engine by Pratt and Whitney [8]. For details of current progress in alloy design, processing technologies, applications and prospects, the reader is referred to references [1,2,9,10,11,12].

TiAl alloys consist at room and service temperature entirely of ordered, intermetallic phases, predominantly of γ-TiAl (L1_0_ structure), α_2_-Ti_3_Al (D0_19_ structure) and β_o_-TiAl (B2 structure). Mainly the inherent brittleness—a result of the intermetallic character of all constituent phases—has made processing a challenging task on the path to industrialization [13]. The combination of thermo-mechanical processing and multiple heat-treatments, which exploit the occurrence of several elapsing phase transformations, has therefore been extensively investigated with the result of enabling the adjustment of different types of microstructures [14]. These in turn allow for tuning of mechanical properties over wide ranges toward the prerequisites of the particular area of operation mainly by the control of morphological parameters, e.g., grain size, colony size and aspect ratio or lamellar interface spacing. In this respect the fully lamellar or nearly fully lamellar microstructures evidence the best combination of strength, creep resistance, ductility, and fracture toughness—all of which are required for feasible manufacturing processes, to achieve balanced mechanical properties as well as to provide structural integrity during a components lifetime [2,14].

**Figure 1:**
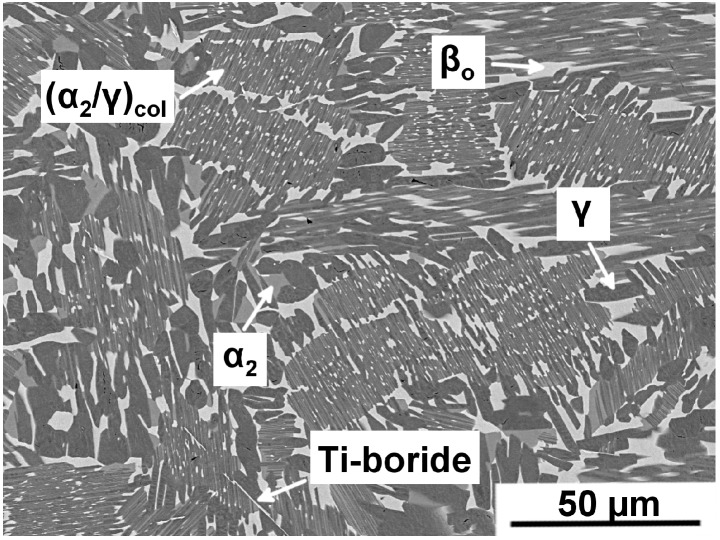
Cast and hot-isostatically pressed (cast/HIP) microstructure of a TNM alloy taken by scanning electron microscopy (SEM) in back-scattered electron (BSE) mode. The microstructure comprises of γ, α_2_, and β_o_ phase of globular morphology as well as of α_2_/γ colonies with β_o_ precipitates in between (secondary precipitates). The γ phase appears dark gray, the α_2_ phase is light gray and the β_o_ phase shows the brightest contrast. Moreover, a rod-shaped Ti-boride is visible at the bottom of this image.

In recent years, a novel subclass of so-called β-solidifying TiAl alloys, the TNM alloy, has emerged [2,12,15,16,17]. This alloy solidifies entirely via the disordered β phase (A2 structure) (L → L + β → β …), which results in an homogeneous, fine grained and almost texture-free microstructure in contrast to peritectically solidifying alloys (L → L + β → α …), which are prone to segregation, coarse microstructure and a strong casting texture [2,12,15]. TNM alloys comprise, next to Ti and Al, the alloying elements Nb, Mo and small amounts of B, and were designed with the aim of providing a forgeable alloy exhibiting balanced mechanical properties that can be attained via near-conventional manufacturing routes and subsequent heat-treatments [12,18,19]. At elevated temperatures this alloy exhibits a sufficient amount of disordered β phase due to the β-stabilizing elements Nb and Mo, which facilitates hot-workability. This phase is, however, undesirable at service temperatures, but can be removed or reduced via ensuing heat-treatments [12,18]. Figure 1 depicts the microstructure of a TNM alloy in the cast and hot-isostatically pressed (HIP) condition taken by scanning electron microscopy (SEM) in back-scattered electron (BSE) mode. In this micrograph, all major constituent phases are visible and are labeled accordingly. The microstructure is homogeneous, i.e., no significant segregation of alloying elements occurred during processing. Boron, which is added to TNM alloys as a grain refining agent, acts as heterogeneous nucleant during solidification and subsequent phase transformations [20]. Thereby, B is mostly present in thermally stable borides—one of which is visible at the bottom of Figure 1. The depicted cast/HIP microstructure corresponds to the starting condition for following forging operations or to adjust the microstructure directly via refined heat-treatment strategies [21]. In order to enhance the high-temperature capability of TNM alloys, alloying with microalloying elements, such as C and Si, is a suitable means [22,23,24,25]. These alloys are then denoted as TNM^+^ alloys and represent a most promising alloy subclass, which effectively increases the maximum operating temperature of TNM alloys.

A minor, but nevertheless important phase, which can hardly be visualized by SEM, due to its extreme fineness, is the ω_o_ phase, which can occur in many TiAl systems including the TNM alloy [26,27]. In these β_o_ phase containing TiAl alloys, ω_o_ particles are observed to precipitate from the β_o_ phase and a simultaneous hardening has been reported [28,29,30,31]. However, details of the occurring precipitation reaction are matter of ongoing scientific issues among scientists. As a simultaneous significant solute redistribution is typically observed, atom probe tomography (APT) corresponds to the method of choice for a detailed investigation [22].

APT (and atom probe field ion microscopy (APFIM)) was extensively applied to many fundamental questions of this alloy class. Two particularities of TiAl alloys account for the intense utilization of APT: (i) mechanical properties, especially ductility, are dependent on the interstitial content of the individual phases [32]; and (ii) as microstructures often consist of extremely fine-scale constituents, high-resolution techniques are required for a detailed characterization [33,34]. The following section will give a brief literature survey encompassing APT studies of the past three decades in the field of titanium aluminides.

Early studies revealed superior properties of α_2_/γ two-phase alloys, especially of lamellar structures, particularly in terms of higher strength levels, ductility and fracture toughness in comparison to single-phase alloys, which are either based on γ or α_2_. Hence, fundamental research aimed at understanding underlying phenomena. These studies revealed that O impurity levels are highest in the α_2_ phase of two-phase alloys [35,36,37]. Its removal from the γ phase was argued to promote ductility by a so-called “scavenging effect”, i.e., a purification effect keeping the γ phase ductile [35,36], or entirely by structural features of the lamellar arrangement [37]. Menand et al. [38] rebutted the “scavenging effect” of the α_2_ phase as they were able to evidence that the γ phase of single-phase and two-phase alloys shows equivalent O concentrations, corresponding to the maximum solubility of this phase. As this solubility level is much lower than the alloy’s impurity contamination, it is argued that even in high purity single-phase γ-TiAl alloys, the O excess leads to fine-scaled oxide precipitates or a local enrichment, which in turn result in embrittlement. Furthermore, Lefebvre et al. [39] evidenced a strong dependence of the maximum interstitial solubility on the off-stoichiometric composition of the γ phase. It is argued that interstitials such as C, N or O prefer to occupy Ti_6_ octahedral sites, which are inherently present in the α_2_ phase but not in the γ phase. However, if the phase composition of the γ phase deviates toward the Al-lean side of the stoichiometric composition, the surplus of Ti atoms occupies Al sites, which in turn results in the creation of Ti_6_ octahedral cavities. This conclusion was later picked up by Scheu et al. [40] to explain the observed increase in C solubility by alloying with Nb. As Nb preferentially substitutes for Ti in the γ phase [41,42], a surplus of Ti is generated, which locates on Al sites, hence, acts equivalently to off-stoichiometry, effectively increasing the concentration of Ti_6_ octahedral sites.

APT was moreover utilized for detailed studies of different alloying concepts mainly in terms of element phase preference and its effect on phase equilibria. TiAl alloys of second and third generation (see references [1,2]) typically contain one or more different transition metal additions. In this respect, Nb, Mo, Ta, W, V, Cr, and Mn have been widely investigated. APT studies of Nb, which is included in most engineering alloys [43,44,45,46,47], revealed only weak partitioning and a homogeneous distribution for both two-phase alloys [48,49,50,51] and three-phase alloys [22,52]. At the same time Mo strongly favors the β_o_ phase in three-phase alloys [22,52]. In this respect Vb elements generally show a weak phase preference for the β_o_ phase [22,48,49,50,51,52], whereas VIb elements generally show a strong phase preference for the β_o_ phase [22,29,52]. The tendency of phase preference corresponds to an increased β-stabilizing effect of VIb elements in comparison to Vb elements [53].

Some alloying elements, moreover, have been shown to effectively increase creep resistance or, more generally, increase the microstructural stability. These effects have been argued to stem from interfacial segregation essentially decreasing interface mobility [54,55]. APT, which is also especially applicable for the study of interfacial segregation due to its superior spatial resolution and the potential of directly quantifying the extent of segregation, evidenced interfacial excesses at phase interfaces of both heavy elements such as W [51,56] and Hf [51] as well as light elements such as C [22].

Progress of recent years in APT technique allows for the generation of reliable data with a reasonable experimental effort. Hence, more sophisticated questions may be answered that arise in current multi-phase multi-component TiAl alloys to improve the existing and to achieve knowledge required for new innovations. Issues of advancing titanium aluminides using APT are characterization of the lamellar structure [51,52], alloying effects [22,40], characterization of ceramic-type strengthening particles, and occurring phase interfaces [57,58] or underlying phenomena of in-service oxygen embrittlement [59], just to name of few examples. This article reviews questions in the development of TiAl alloys that were successfully answered using APT and highlights possibilities of this technique. In particular fundamental questions as well as issues pertaining to the enhancement of TNM alloys are contemplated in the frame of complimentary methods.

## 2. Atom Probe Tomography

The basis of APT has been established in several textbooks and reviews [60,61,62]. Nevertheless, this section should give a short overview of the fundamentals of this technique, the progress that resulted in the state-of-the-art methods as well as the preparation techniques suitable for the specimen production of TiAl alloys.

### 2.1. Background of Technique: Principles of Method and Compositional Measurements

APT is based on the field evaporation of surface atoms near the tip of a needle-shaped specimen, whereby both time-of-flight mass spectroscopy and position sensitive ion detection are utilized [61,62]. Specimens need to be needle-shaped with tip radii of ≈20–100 nm in order to reach sufficient local electric fields of ≈10–60 V/nm resulting in ionization and emission of surface atoms at cryogenic temperatures and ultra-high vacuum. An approximation of the electric field occurring in the vicinity of an approximately spherical tip is given by Equation (1):

(1) $$F\;=\;\frac{V}{k_{f}\cdot R}$$

where *F* is the electric field strength induced at the tip’s apex with a radius of curvature *R* and *k_f_* denotes the field factor, which includes the tip shape as well as the electrostatic environment, i.e., corresponds to a geometrical correction stemming from specimen and mounting geometry and *V* corresponds to the voltage applied [61,62]. From Equation (1), one can recognize the requirement of the specimen’s tip to be extremely sharp due to naturally given upper limits of voltage. During the evaporation process, atoms that are positioned close to steps are more likely to evaporate resulting in a self-regulation of the specimen geometry. The evaporated ions follow the electric field lines created between the specimen and the detector. The evaporation process is controlled concomitantly to the measure of the time of departure by creation of a standing field below the evaporation field of the atoms of the material investigated. This field is then superimposed either by voltage pulses or laser (thermal) pulses—both of which result in a controlled emission of ions. These short duration pulses allow for a distinct discrimination of the time-of-flight of each ion that hits the position sensitive detector. The information gained is the mass-to-charge ratio for each evaporated ion. Data post-processing such as background subtraction or peak deconvolution is often required to identify the chemical identity of a particular species and, thereby, create an accurate chemical analysis. Moreover, the three-dimensional specimen volume can be reconstructed using the information determined by the position sensitive detector providing knowledge of the spatial distribution of the atoms in, e.g., phases or clusters in a very descriptive way [61].

### 2.2. The Local Electrode Atom Probe

**Figure 2:**
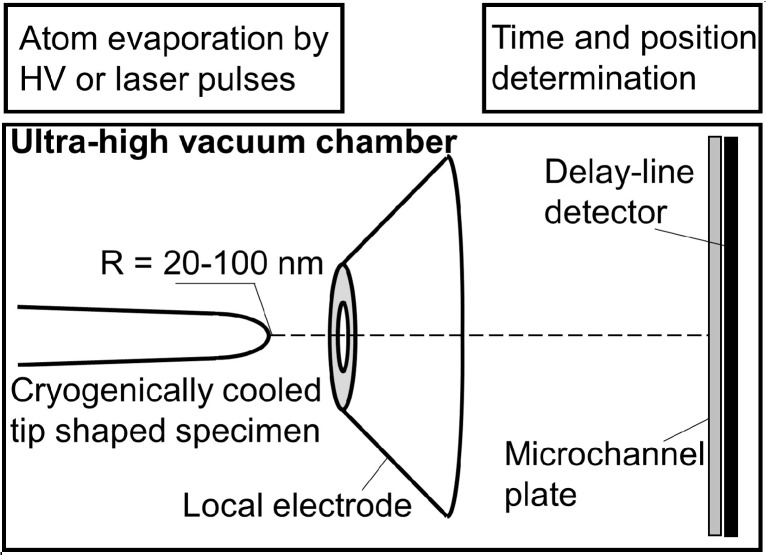
Scheme of the major components present in a local electrode atom probe (LEAP) redrawn after reference [62]. Surface atoms near the tip’s apex are evaporated either by high voltage or laser pulses and accelerated by standing voltage toward the position sensitive detector. Time-of-flight mass spectroscopy allows for identification of the chemical identity; position determination enables a three-dimensional reconstruction of the measured specimen volume.

The introduction of the local electrode atom probe (LEAP™) [63,64] has allowed for a widened utilization of APT and much work has been done since. Recent advances of method and data evaluation are explained in detail in references [64,65,66,67,68,69]. In the LEAP™ instrument a standing voltage is applied between the specimen and the counter electrode, which is, as in the case of any atom probe, superimposed by high voltage or laser pulses. The novelty is the introduction of the local electrode, whereby the specimen is positioned very close to this electrode as schematically depicted in Figure 2. This experimental arrangement allows for a dramatic increase of measureable sample volumes and an extended field of view. Evaporated ions travel through a hole in the local electrode toward the position sensitive detection system, whereby the generated signal as well as its evaluation and interpretation may be performed equivalently as described in Section 2.1.

Further enhancement of mass resolution may be attained via utilization of a reflectron belonging to the so-called techniques of energy compensation or flight time compensation. To this end, the travelling ions enter an electrostatic field and are, thereby, deflected onto a curved pathway. Ions of lower energy will be deflected first and, hence, travel a shorter path than ions of higher energy. A longer path increases the time-of-flight and vice versa, i.e., the peak width in the time-of-flight spectrum is narrowed corresponding to an increased mass resolution potential. However, one major drawback of the reflectron system is the concomitant reduction of detection efficiency.

### 2.3. Methods of Specimen Preparation

**Figure 3:**
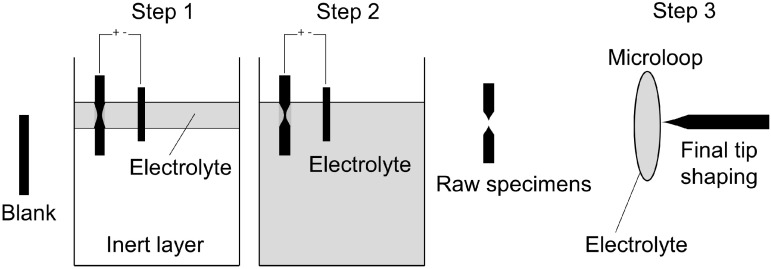
Scheme of the specimen preparation routine by electropolishing. The sample material is cut into prismatic blanks and mounted into Cu crimps. Subsequently, these blanks are etched and sharpened using three steps, the first two resulting in a raw tip-shaped geometry, which has, however, to be further sharpened using a microloop procedure denoted as Step 3. Drawn after references [60,61].

There exist two distinct preparation routes that are suitable and necessary for the study of TiAl alloys by means of APT. The first approach follows a sequence that is schematically depicted in Figure 3. Thereby, blanks possessing ≈1–2 cm length and a quadratic cross-section of ≈300 µm side length are usually cut using precision cutting tools. These are then mounted into Cu (or Ni) crimps and are etched in a first step (Figure 3, Step 1) using a combination of electrolyte and inert liquid to create a neck roughly in the middle of the specimen. Subsequently, the inert layer is removed and the specimen is etched until it fractures (Step 2). Following this procedure, final shaping and sharpening is conducted using a microloop with a drop of electrolyte (Step 3). For TiAl alloys an etchant consisting of 5% perchloric acid in acetic acid has been successfully used for Steps 1 and 2 using polishing voltages of ≈25 V and ≈15 V, respectively. The best results of final tip shaping were obtained using 2% perchloric acid in butoxyethanol at ≈10 V. For further details on electropolishing routines and other preparation methods the reader is referred to the textbooks [60,61].

**Figure 4:**
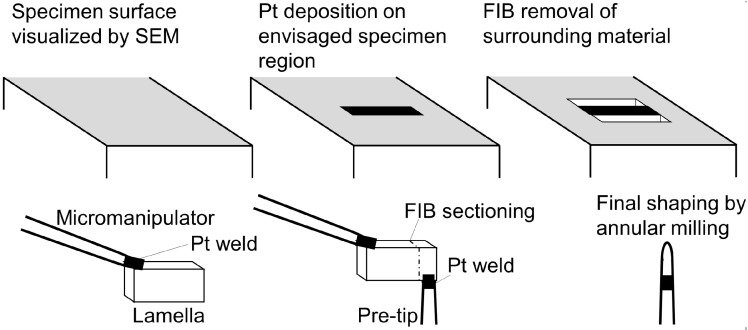
Scheme of the atom probe tomography (APT) specimen preparation by lift-out technique. In the first step a region of interest is identified using SEM and subsequently protected from Ga implantation by a Pt deposit. Contiguous material is then excavated at both sides generating a lamella, which can then be removed using a micromanipulator. Following this sequence, parts of the lamella can be transferred to any kind of suitable pre-tip. Once mounted, the final tip shape can be obtained by annular milling. Schematically redrawn according to references [70,71].

APT specimens can be prepared alternatively using the focused ion beam (FIB) based lift-out technique as described in detail in references [70,71,72,73,74,75]. This procedure is usually carried out in a dual beam FIB device equipped with both an electron as well as an ion source. The sequence of steps conducted is represented schematically in Figure 4. In a first step the SEM is simply used to identify a region of interest for the following measurement. Subsequently, the material is protected from Ga implantation and concomitant sample contamination and amorphization by a Pt deposit. A lamella is then formed upon excavation at two sides contiguous to the Pt deposit using the ion beam. The lamella can then be lifted out after cutting free using a micromanipulator and can be mounted to any kind of pre-tip. This can be the posts of a Si needle pad, tips generated on a cut and electropolished transmission electron microscopy (TEM) grid [73,75,76] or a single tip. At last the final tip shape is generated using annular milling, whereby voltage and current are gradually reduced to decrease Ga contamination [77].

## 3. Effects of Alloying Elements

### 3.1. Niobium and Molybdenum: Phase Formation, Phase Preference and Phase Stability

This section will focus on the effects of the decisive substitutional alloying elements that are typically used in advanced TiAl alloys as studied by APT and will view them in the context of established alloying related effects. Especially, different transition metals have been identified as valuable additions to increase both ductility and strength. For example V, Cr, Mn, Nb, Mo, Ta, and W are all among most common additions to TiAl alloys [15,17,53,78]. However, effects of Nb and Mo are of particular importance for the understanding of the TNM alloy and related alloys as they constitute the major alloying elements [79,80,81].

**Figure 5:**
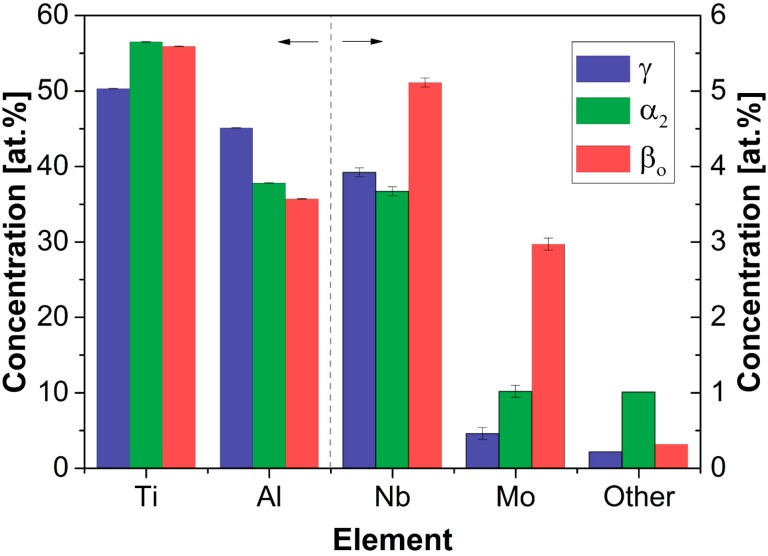
Phase composition of a TNM alloy in the homogenized condition. Concentrations of the major alloying elements, Ti and Al, are displayed on the left ordinate, whereas concentrations of the minor alloying elements, Nb, Mo as well as impurities denoted as “Other” are given on the right ordinate. Titanium shows a slightly higher concentration in α_2_ and β_o_ than in the γ phase and at the same time the Al concentration is reduced. This enormous difference in phase composition in comparison to the ideal binary phases (Ti_3_Al and TiAl, respectively) is due to preferred lattice site occupation of transition metal atoms on Ti sites in the α_2_ phase and Al sites in the β_o_ phase. Nb and Mo both show a tendency to accumulate in the β_o_ phase corresponding to their well-established β-stabilizing effect, which is more pronounced in case of Mo. Impurities, mainly constituted by interstitial elements like C, N or O (here their sum is referred to as “Other”), prefer to locate in the α_2_ phase.

Niobium has been identified as a crucial element for the enhancement of high-temperature strength [47,82,83] and oxidation resistance [43]. The strengthening effect observed in two-phase alloys (comprising of α_2_ and γ phase) has been recognized to stem mainly from an increasing amount of α_2_ and from an occurring structural refinement [84]. In the case of β-solidifying alloys, β_o_ phase is retained to room temperature, which is due to the fact that Nb acts as a β-stabilizing element. Hence, the Nb distribution in the constituent phases is expected to differ from classic two-phase TiAl alloys and is, thus, of particular interest as strong partitioning potentially results in microsegregation, i.e., in microstructural inhomogeneities, that could deteriorate creep and fatigue resistance [83]. Therefore, the local chemical composition of the individual phases of a TNM alloy in the homogenized condition was determined by APT, as reported in reference [22]. Chemical compositions are depicted in Figure 5 in the form of a bar chart, neglecting for the moment the occurrence of a minor phase fraction of ω_o_ phase, which will be dealt with in Section 3.3. Considering Ti and Al, it is of particular interest to note that the composition of the phases α_2_ and β_o_ deviates significantly from the compositions of the ideal binary phases (Ti_3_Al and TiAl, respectively). This strong deviation of both phases is due to differing site occupation behavior of the transition metal atoms that are alloyed in the TNM system. While all transition metals prefer to substitute at Ti sites in the α_2_ phase, there is a strong trend for the substitution at Al sites in the β_o_ phase, as suggested by ab initio calculations in reference [85]. Niobium shows a similar distribution between the individual phases with a weak preference for the β_o_ phase only. This observation is in agreement to the fact that Nb was identified as a β-stabilizing element [2,53]. However, although its addition results in the introduction of the β_o_ phase, which is stable at room and service temperature, there are still significant amounts dissolved in the α_2_ and γ phase, i.e., the enrichment within the β_o_ phase does not entirely remove Nb from the other phases. In contrast, Mo strongly partitions between the phases, whereby the β_o_ phase is most favored. This observation corresponds well to the fact that Mo has been reported to be a by far stronger β-stabilizing element than Nb [2,53]. This effect has far-reaching implications on processing, e.g., on the hot-workability. Since additions of Mo, already in modest amounts, stabilizes the disordered β phase at elevated temperatures, hot-working becomes possible using near conventional hot-working equipment [18,19]. Most importantly, the volume fraction of β/β_o_ phase can be reduced significantly on ensuing heat-treatment as its presence deteriorates the creep resistance [86,87]. This is only possible due to the particular curve progression of phase fractions occurring in the TNM system, i.e., the course of the β phase fraction shows a pronounced C-shape with a minimum between 1250 °C and 1280 °C [12,88]. Using a second heat-treatment step, which is typically conducted slightly above the envisaged service temperature, the amount of β phase can be further reduced due to the fact that the stabilization of the β phase by β-stabilizing elements diminishes in the course of the ordering reaction, i.e., Mo strongly stabilizes the disordered β phase, but hardly stabilizes its ordered counterpart β_o_ [80]. Moreover, a fine lamellar structure is adjusted and the phase fractions are equilibrated, yielding a reduced contribution of phase transformation to the creep elongation [2,18].

Impurity levels are most pronounced in the α_2_ phase as discernible from Figure 5, where the sum of all impurities present in this alloy is shown. This observation is due to the fact that most impurities correspond to interstitial elements, which are mostly dissolved in the α_2_ phase due to the inherent presence of Ti_6_ octahedral sites, where interstitials are incorporated favorably. Both other phases do not constitute any of these sites intrinsically. Some other impurities, however, corresponding to Fe, Cr and V, as identified by APT, all of which represent typical β-stabilizing elements, contribute to the increased impurity levels present in the β_o_ phase in comparison to the γ phase.

### 3.2. Boron Addition and Boride Formation

Boron has been identified as a particularly valuable alloying element in TiAl alloys [20,89]. Early studies revealed a significant grain refinement effect, which was attributed to the presence of thermally stable borides that act as heterogeneous nucleation sites during solidification and on ensuing phase transformation [89]. Some details about the grain refinement effects, especially, as they depend on the evolving phase transformation sequence, i.e., refining effects differ from β-solidifying alloys to peritectic alloys and the cooling rate applied, remain, however, matter of debate and ongoing research [90]. A detailed analysis of the chemical composition of these borides can help to understand, e.g., effects of alloying elements on their stability. Larson et al. [91] determined chemical compositions of different Ti-borides occurring in a Ti-47Al-2Cr-2Nb-0.15B (in atom%, at%) (TIA-20) alloy using APFIM. Results of a measured mono-boride are reproduced in Table 1 and are compared to the chemical composition we have determined for a boride present in the TNM alloy as visible in Figure 1. In our case, the investigated specimen was prepared by site-specific specimen preparation using the FIB technique. Apparently, according to the evaluated chemical composition, this boride similarly corresponds to a mono-boride. Interestingly, the boride contained in the TNM alloy exhibits a significant and by far greater amount of Nb than in the TIA-20 alloy and also some Mo. The boride observed in the TNM alloy, however, contains ≈45 at% B only, i.e., apparently its composition is off-stoichiometric either by creation of anti-site defects or vacancies possibly due to the alloyed elements. The presented results, moreover, evidence that Ti-borides occurring in TiAl alloys show hardly any solubility for Al and are comprised mainly of Ti and B.

### 3.3. The Formation of ω_o_ Phase

**Figure 6:**
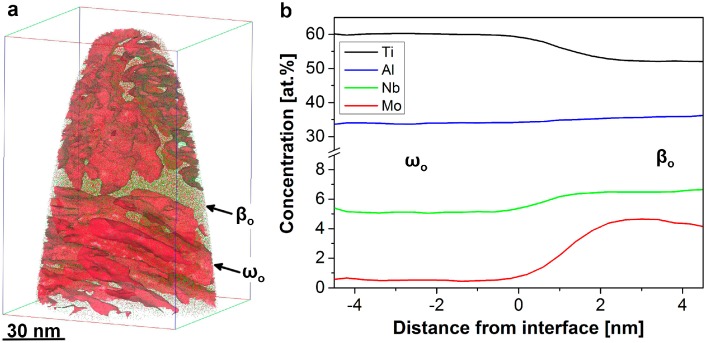
APT reconstruction containing β_o_ and ω_o_ phase and proximity histogram. (**a**) Only Nb (green) and Mo atoms (red) are indicated for clarity. Moreover, isoconcentration surfaces have been generated at 1.3 at% Mo. These interfaces clearly separate regions of β_o_ and ω_o_ phase. ω_o_ precipitates show parallel arrangements, which correspond to the predominating orientation relationship (OR) (see text); (**b**) Concentration profiles calculated as proximity histograms across the interfaces depicted in (**a**) are shown. The concentration of Al remains nearly unaltered, whereas Mo and Nb on the one hand and Ti on the other hand show an opposing partitioning behavior.

As mentioned previously most engineering TiAl alloys that are currently in use are alloyed with modest to high amounts of Nb [46,82], which is generally vital for high-temperature strength as well as creep and oxidation resistance. The introduction of this element, however, results in an increased susceptibility to the formation of additional phases such as the ω_o_ phase [26,27,28,29,31], which was observed to result in an increasing hardness [22,28]. In the case of β-solidifying TiAl alloys, this phase precipitates from the β_o_ phase at temperatures below ≈825 °C [28] in contrast to alloys free of β_o_ phase, where precipitation from the α_2_ phase can occur [92,93]. For the detailed understanding of the precipitation process occurring in TNM alloys, it is of particular interest to understand how the elements redistribute between β_o_ and ω_o_ phase during the precipitation sequence and, thereby, withdraw information pertaining to stabilization and destabilization. Hence, APT specimens of a heat-treated TNM sample containing β_o_ and ω_o_ phase were prepared site-specifically as described in reference [75]. There, a TNM specimen that was annealed above the ω_o_-solvus temperature and cooled slowly in the furnace, allowing for the precipitation reaction to take place, as reported in reference [22]. The presence of both β_o_ and ω_o_ phase was verified by complimentary transmission Kikuchi diffraction conducted directly at the site-specifically prepared APT [22,75]. An APT reconstruction containing the alloying elements Nb (green line) and Mo (red line) is shown in Figure 6a, where isoconcentration surfaces at 1.3 at% Mo are also indicated. A clearly inhomogeneous distribution of these elements is discernable and the phase decomposition occurring can be visualized explicitly by the isoconcentration surfaces. As these surfaces are created with the aid of a concentration gradient it should be noted that they do not necessarily coincide the actual crystallographic phase boundaries. Some of the ω_o_ particles show a parallel arrangement elongated along preferred growth directions that correspond to the established orientation relationship (OR) ${\langle 111\rangle }_{\beta_{o}}{\left\{1\overset{-}{1}0\right\}}_{\beta_{o}}∥{\left[0001\right]}_{\omega_{o}}{\left\{11\overset{-}{2}0\right\}}_{\omega_{o}}$ [94].

For a quantification of the changes in chemical composition across the interfaces of Figure 6a, proximity histograms of the major alloying elements Ti, Al, Nb, and Mo were calculated and are depicted in Figure 6b. The phase boundary appears to be chemically diffuse, while being crystallographically sharp. The Al distribution seems nearly homogeneous, whereas Nb and Mo show opposing partitioning in comparison to Ti. The Mo redistribution occurring during growth of the ω_o_ particles is clearly the most pronounced effect and is indicative for an actual phase decomposition. Apparently, Mo is rejected from the ω_o_ phase and enriched in the β_o_ matrix in agreement with references [22,28]. In the vicinity of the interface, a Mo pile-up occurs as visible in Figure 6b. The appearing diffusional redistribution is presumed to limit the formation kinetics.

### 3.4. Enhancement of the TNM Alloying Concept: Effects of Carbon Addition

**Figure 7:**
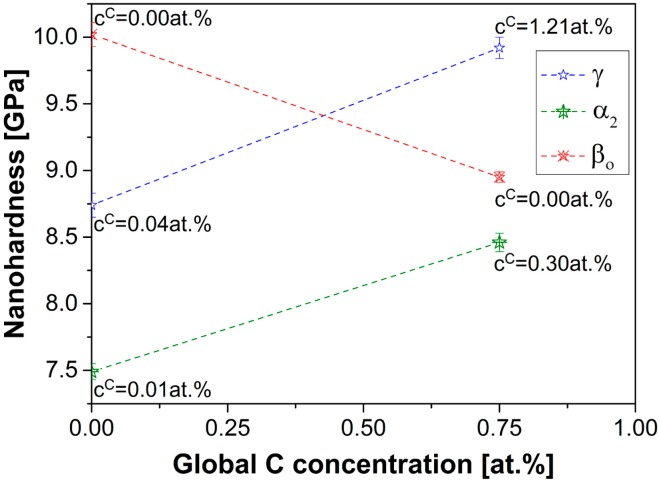
Hardness as measured by nanoindentation of the individual phases of a TNM alloy and a C-containing derivative, TNM0.75C. The error bars indicated correspond to the standard errors of the mean. In the C-free state the β_o_ phase is the hardest phase followed by α_2_ and γ. By alloying with C, the latter two phases harden via solid solution hardening, whereas the β_o_ phase softens due to the absence of ω_o_ particles. The C concentration of each individual phase of both alloys as measured by APT is labeled next to the respective hardness values. Dashed lines are indicated only to guide the eye.

The enhancement of high-temperature strength has constantly been a goal in the design of novel TiAl alloys. In this regard particularly alloying with the element C has been investigated. This interstitial element strengthens the material either by solid solution hardening or by carbide precipitation [32,95,96]. The predominating mechanism is, thereby, determined by the alloy composition and the materials’ thermal history. Carbon is mainly located in the direct environment of six Ti atoms, i.e., in so-called Ti_6_ octahedral cavities, as suggested in references [38,39]. Following this train of thought, the α_2_ phase is argued to show the highest intrinsic solubility as it is the only phase in the compositional range of engineering TiAl alloys that intrinsically possesses this type of cavities, whereas the both other phases, γ and β_o_, do not possess any. In case of the γ phase a strong dependence of the maximum solubility for C is observed with respect to the Al concentration. In particular, toward the Al-lean off-stoichiometric composition, the C solubility increases [39]. This observation is justified by the argument that the excess of Ti atoms tend to occupy Al sites and, thereby, introduce Ti_6_ octahedral cavities that can be occupied by C. Similarly, the addition of alloying elements, especially transition metal additions such as Nb, increases the solubility limit for interstitials in the γ phase [40]. The mechanism is contended to be related to the fact that Nb preferentially occupies Ti sites [41,42] in the L1_0_ lattice and, thus, similarly as in the case of off-stoichiometry introduces a surplus of Ti atoms, which relocate to the Al sites, whereby also similarly favorable cavities are created. In case of the β_o_ phase there are no favorable sites present in the stoichiometric B2 structure, which can, however, not be introduced by substitution, as all transition metal atoms preferentially occupy Al sites [85]. Therefore, the β_o_ phase is expected to show a very limited C solubility. In order to verify these theoretical considerations, the chemical composition of the individual phases of a model TNM alloy that was additionally alloyed with 0.75 at% C (TNM0.75C) was determined by APT in the homogenized condition after forging as reported in references [22,97]. In agreement with the theory on C distribution, the C concentrations were found to follow the sequence $c_{\alpha_{2}}^{C}\;>\;c_{\gamma }^{C}\;>\;c_{\beta_{o}}^{C}$ as indicated in Figure 7.

Moreover, in this figure the local mechanical properties of the constituting phases as determined by nanoindentation are correlated with the global C concentration [22]. Nanoindentation is, in this respect, a particularly suitable method as it allows withdrawing information about mechanical properties of individual microstructural constituents, which can be correlated to the local chemical compositions analyzed by APT. Apparently, α_2_ and γ show an increase in nano-hardness by the incorporation of C, which can be explained by a solid solution hardening mechanism as no carbides were observed in the investigated material condition. This effect is in agreement to the C concentrations determined by APT. The nano-hardness of the β_o_ phase, however, decreases with increasing global C content, which is interesting to note, as the β_o_ phase does not show any solubility for C. It has been evidenced in the literature that C is a strong stabilizer of the α_2_ phase, while the phase fraction of β_o_ is reduced by its incorporation [23]. As mentioned before, Mo tends to partition strongly to the β_o_ phase, i.e., if the β_o_ phase fraction is reduced, the amount of Mo in this phase increases. An increasing amount of Mo tends to inhibit the formation of ω_o_ phase as discussed in Section 3.3. The absence of ω_o_ particles in the interior of the β_o_ phase of the C-containing alloy, results in a reduced nano-hardness as no precipitation hardening contributes [28].

**Figure 8:**
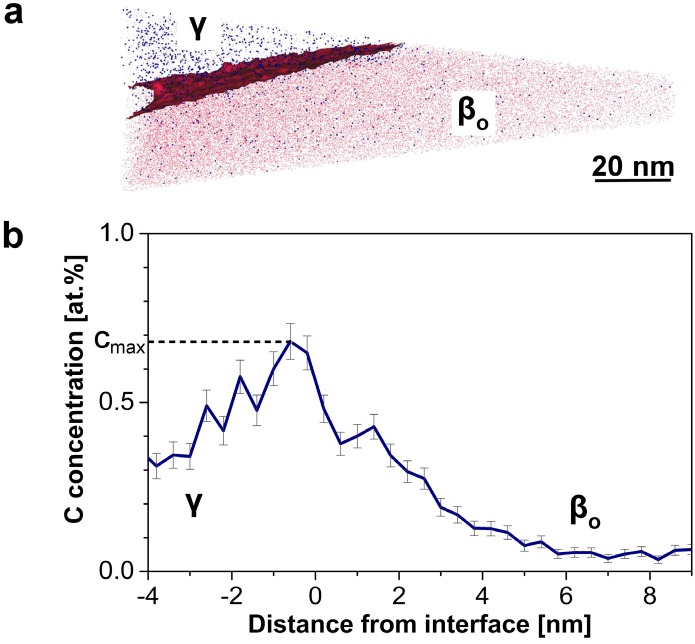
(**a**) APT reconstruction of a TNM0.75C specimen containing a β_o_/γ interface; and (**b**) C proximity histogram across this interface. (**a**) A clearly inhomogeneous distribution of the alloying elements Mo (red) and C (blue) across the interface is visible. The interface indicated corresponds to a Mo isoconcentration surface, which shows the most pronounced concentration gradient. The histogram in (**b**) evidences significant segregation of C to the β_o_/γ interface, with a local maximum C concentration of ≈0.7 at%. (Reprinted with permission from [22], Copyright© 2015, Elsevier).

Research on C-containing TiAl alloys has, moreover, evidenced the significant impact of C on the microstructural stability in terms of resistance against discontinuous precipitation of lamellar structures [24] as well as reduced recrystallization kinetics after hot-working [98]. These effects, on the one hand, are due to the impaired redistribution of the strongly partitioning element C required for the formation of new grains during any kind of recrystallization. On the other hand, solute-drag effects may be responsible for a reduced interfacial mobility [99]. In order to visualize and quantify the presence and extent of such segregation effects, APT specimens were prepared site-specifically using a dual beam FIB as reported in references [22,75]. A reconstruction and a proximity histogram of a TNM0.75C specimen are represented in Figure 8. In this case the tip contains a β_o_/γ interface and only the minor alloying elements Mo (in red) and C (in blue) are depicted. Obviously, an inhomogeneous distribution is evident in Figure 8a corresponding to prevailing strong partitioning. The proximity histogram calculated across this interface demonstrates quantitatively significant local C enrichment in the vicinity of the interface with a local maximum C concentration of ≈0.7 at%. This enrichment exemplifies clearly that interfacial segregation of C is existent, which in turn increases microstructural stability of TNM alloys, when alloyed with C [23,24].

## 4. Characterization of Nano-Scaled Lamellar Structures: Effects of Silicon Addition

**Figure 9:**
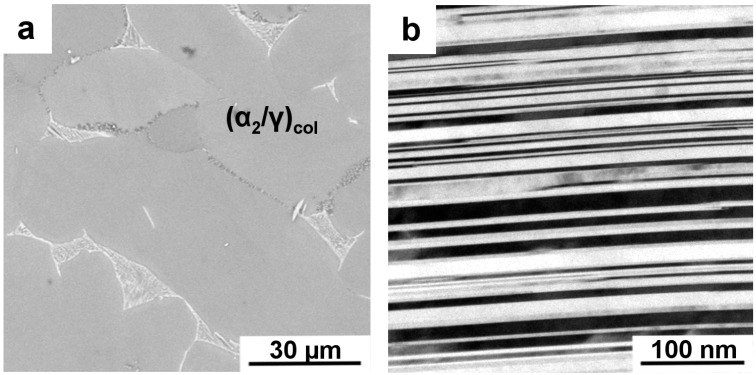
Microstructure after two-step heat-treatment taken by (**a**) SEM in BSE mode and (**b**) transmission electron miscroscopy (TEM) bright field image, as reported in reference [52]. Prior supersaturated α_2_ grains have decomposed to α_2_/γ colonies, which cannot be resolved using SEM. Lamellae of each grain show only one orientation variant according to the Blackburn OR. Between lamellar colonies β_o_ phase and several transformation products are visible (see text). The nano-lamellar structure can be evidenced by TEM (**b**), where the specimen has been tilted into edge-on condition.

A further crucial alloying element that is often added to TiAl alloys is Si. Although its effect is controversially discussed in the literature, the general consensus points toward an increase in creep resistance via stabilization of the microstructure [100,101,102]. In particular, the precipitation of ζ-silicides (Ti_5_Si_3_) at lamellar interfaces is observed frequently in many alloy systems, which is argued to stabilize these interfaces as well as to reduce dislocation mobility. The specific precipitation mechanism, which ensues especially in two-phase alloys with a low α_2_ volume fraction due to heat-treatment and creep, leads to aligned particles at lamellar interfaces [103]. The α_2_ phase contains the most Si, while the solubility for Si is rather limited in the γ phase. Hence, silicides precipitate once the α_2_ phase fraction reaches a lower margin, which restricts also the global solubility for Si. However, advanced engineering TiAl alloys, such as the TNM alloy, typically contain higher amounts of α_2_ phase and, furthermore, a varying amount of β_o_ phase—both of which may alter to evolving reaction sequence. Therefore, a nano-lamellar structure was generated via a two-step heat-treatment in a TNM alloy containing Si as reported in reference [52]. During the first heat-treatment step, the solution heat-treatment conducted above the γ-solvus temperature, only disordered α and β phases are present [88,104]. On subsequent rapid cooling lamellar γ phase formation is suppressed, which can be finely and homogeneously precipitated on ensuing annealing as demonstrated in references [105,106,107]. The annealing procedure is, thereby, typically conducted slightly above the envisaged service temperature. During this stage γ lamellae form in the supersaturated α_2_ grains upon sufficient thermal activation by the creation of two Shockley partial dislocations bordering a stacking fault that locally accomplishes the change in crystal structure [108,109,110]. Figure 9 depicts such a structure generated in a TNM^+^ alloy (Ti-43.5Al-4Nb-1Mo-0.1B-0.3C-0.3Si in at%) after heat-treatment at 1340 °C for 15 min followed by oil quenching and subsequent annealing at 800 °C followed by furnace cooling. In Figure 9a a SEM micrograph is shown, but the ultra-fine lamellar structure within the α_2_/γ colonies cannot be resolved. Between these colonies the prior β_o_ phase is visible, which decomposed during annealing into γ platelets, ω_o_ particles and remaining β_o_ phase. The lamellar structure can be resolved by TEM as depicted in Figure 9b. The interfaces are clearly delineated and appear smooth without significant interfacial roughness. All γ lamellae of a prior α_2_ grain are aligned parallel due to the Blackburn OR [108]. Details regarding the mechanism of lamellar structure formation can be found in references [33,109,110]. Most importantly, no ζ-type precipitates are observed in the vicinity of the lamellar interfaces.

**Figure 10:**
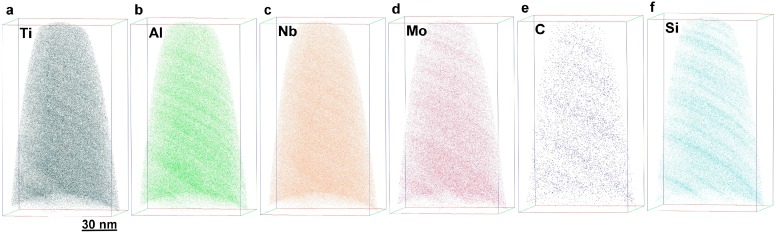
Reconstructions of the lamellar structure of a TNM^+^ alloy. In all images, the lamellar structure is visible to different extents corresponding to different partitioning ratios. Carbon shows a tendency to accumulate in the α_2_ phase and Si partitions even more pronounced to this phase. See Table 2 for quantitative analysis.

APT reconstructions of the individual alloying elements corresponding to the lamellar structure depicted in Figure 9 are shown in Figure 10. In each image, the lamellar structure is apparent to a greater or lesser extent, corresponding to their partitioning between the α_2_ and γ lamellae. The partitioning of C and Si toward the α_2_ phase is particularly distinct with Si showing a more pronounced tendency to partition to this phase, which is in agreement with reported literature data [111,112] (see Section 3.4 for a more detailed discussion of the accumulation of C in the α_2_ phase). Although these elements show an isovalent electronic structure, the dominating mechanism must be different as C is dissolved interstitially, while Si is dissolved substitutionally as suggested in references [52,85] by ab initio calculations.

The chemical composition of the lamellar structure depicted in Figure 10 was quantified (Table 2) after separation of the adjacent phases by isoconcentration surfaces defined at a Ti value of 55 at%. Subsequently, the compositional evaluation was conducted by peak deconvolution, which is required due to overlaps in the mass spectrum by different chemical species due to the multi-component nature of the TiAl alloy investigated. The chemical composition of the γ phase is comparably close to its equilibrium composition. However, a deficiency of Al is observed, which is interesting to note as Nb and Mo preferentially substitute for Ti [41,85]. It is, however, energetically favorable to substitute for Ti, which in turn relocates to Al sites as recently shown by ab initio calculations [85]. In case of the α_2_ phase the off-stoichiometry is even more pronounced, i.e., the Ti concentration is reduced and the Al concentration is increased. Apparently, the surplus of Al occupies Ti sites and equivalently do the alloyed transition metal atoms in agreement with the data presented in Figure 5 and reference [85]. Nb shows in the lamellar structure a weak preference for the γ phase, which has similarly been reported for the lamellar structure of two-phase alloys [51]. The opposite is occurring in case of Mo. As mentioned before both C and Si prefer to locate in the α_2_ phase. In case of C this is due to the fact that this phase intrinsically possesses Ti_6_ octahedral sites. Apparently, in case of Si the chemical environment and crystal structure of α_2_ results in an energetically preferred environment, where it preferentially substitutes for Al [85]. These chemical details demonstrate the origin of the absence of ζ-silicides in TNM^+^ alloys in the material condition investigated as suggested by Klein et al. [52]. The α_2_ phase is capable of storing substantial amounts of Si and, therefore, dominates the global solubility limit. TNM alloys contain substantial amounts of α_2_ phase due to lower amounts of Al in comparison to classic TiAl alloys. Hence, all Si present in the interior of each α_2_/γ colony is dissolved and no interfacial precipitation occurs.

## 5. Conclusions

Intermetallic materials based on γ-TiAl have become an engineering material class in the last years and are now increasingly incorporated into novel propulsion concepts in jet and automotive engines. Especially β-solidifying alloys, such as the TNM alloy, have shown to be processible using robust production routes and to achieve reasonable mechanical properties. APT has played a pivotal role in the understanding and the in-depth analysis of these alloys, which has been summarized in this work. APT has been shown to be a powerful characterization tool shedding light upon chemical phase composition, chemical fluctuations, i.e., early stages of precipitation by compositional clustering, partitioning behavior of elements as well as interface characteristics. These phenomena can often solely be analyzed when APT is employed. Thereby, valuable information for the understanding and enhancement of γ-TiAl based alloys is generated. In this work, major findings using APT in correlation with other techniques have been reviewed in the context of existing literature. The major conclusions can be summarized as follows:

- The chemical composition of all constituent phases (γ, α_2_, β_o_) of a TNM alloy was determined. Preferential partitioning of Nb and Mo to the β_o_ phase is discernable and the α_2_ phase shows the highest impurity levels. The element B is mainly present in mono-borides.
- Within the β_o_ phase the precipitation of a further minor phase, the so-called ω_o_ phase, can be detected, which is depleted in the strong β-stabilizing element Mo.
- Carbon, if alloyed to TNM alloys, preferentially resides in the α_2_ phase, followed by the γ phase. The β_o_ phase appears to be completely depleted of C. In the microstructural condition investigated both α_2_ and γ phase are significantly strengthened by solid solution hardening due to the presence of the interstitial element C. However, the β_o_ phase seems to soften by the addition of C, which stems from the fact that the presence of C yields a lower β_o_ phase fraction, thereby increasing the Mo content in this phase, which in turn results in the suppression of the ω_o_ phase formation and its concomitant hardening effects. Carbon, moreover, tends to segregate to interfaces, which may result in lower interface mobilities, thus, stabilizing the microstructure.
- In ultra-fine lamellar structures, Si preferentially occupies the α_2_ phase, whereby neither interfacial segregation nor interfacial precipitation is observed in TNM^+^ alloys, in contrast to other TiAl alloying systems, which is due to a substantial fraction of α_2_ phase where Si is substitutionally dissolved.

## Figures and Tables

**Table 1:** Boride composition determined as reported in reference [91] and in the present work. The standard error is given next to each concentration. Both particles correspond to mono-borides in accordance with a MB stoichiometry, where M represents the constituting metal atoms.

Alloy	Element Concentration (at%)					
	B	Ti	Al	Nb	Mo	Other
TIA-20 [91] ^1^	52.0 ± 3.2	46.3 ± 3.2	0.4 ± 0.4	1.2 ± 0.7	-	0.1
TNM (present study)	45.1 ± 0.01	47.0 ± 0.01	-	6.8 ± 0.03	1.1 ± 0.04	0.0

^1^ Actual alloy composition: Ti-47Al-2Cr-2Nb-0.15B (at%).

**Table 2:** Phase composition of the lamellar structure depicted in Figure 10 as studied in reference [52]. The errors given correspond to the standard errors. Nb shows a weak preference for the γ phase, whereas Mo evidences a weak preference for the α_2_ phase. C and Si prefer to locate in the α_2_ phase.

Phase	Element Concentration (at%)						
	Ti	Al	Nb	Mo	C	Si	Other
γ	49.8 ± 0.02	44.8 ± 0.02	4.01 ± 0.03	0.85 ± 0.03	0.16 ± 0.03	0.21 ± 0.02	0.17
α_2_	61.4 ± 0.03	32.1 ± 0.05	3.90 ± 0.05	1.14 ± 0.06	0.35 ± 0.06	0.51 ± 0.06	0.60
